# Insights from telesurgery expert conference on recent clinical experience and current status of remote surgery

**DOI:** 10.1007/s11701-024-01984-w

**Published:** 2024-06-04

**Authors:** Bernardo Rocco, Marcio Covas Moschovas, Shady Saikali, Giorgia Gaia, Vipul Patel, Maria Chiara Sighinolfi

**Affiliations:** 1 Urology Unit, ASST Santi Paolo and Carlo, University of Milan, via Rudini 8, Milan, Italy; 2 Gynecology Unit, ASST Santi Paolo and Carlo, University of Milan, Milan, Italy; 3 Adventhealth, Global Robotic Institute, Celebration, FL 34747 USA

**Keywords:** Remote surgery, Robotic surgery, Telesurgery, Network structure, Latency

## Abstract

Remote surgery provides opportunity for enhanced surgical capabilities, wider healthcare reach, and potentially improved patient outcomes. The network reliability is the foundation of successful implementation of telesurgery. It relies on a robust, high-speed communication network, with ultra-low latency. Significant lag has been shown to endanger precision and safety. Furthermore, the full-fledged adoption of telerobotics demands careful consideration of ethical challenges too. A deep insight into these issues has been investigated during the first Telesurgery Consensus Conference that took place in Orlando, Florida, USA, on the 3rd and 4th of February, 2024. During the Conference, the state of the art of remote surgery has been reported from robotic systems displaying telesurgery potential. The Hinotori, a robotic-assisted surgery platform developed by Medicaroid, experienced remote surgery as pre-clinical testing only; the Edge Medical Company, Shenzen, China, reported more than one hundred animal and 30 live human surgeries; the KanGuo reported human telesurgical cases performed with distances more than 3000 km; the Microport, China, collected more than 100 human operations at a distance up to 5000 km. Though, several issues—cybersecurity, data privacy, technical malfunctions — are yet to be addressed before a successful telesurgery implementation. Expanding the discussion to encompass ethical, financial, regulatory, and legal considerations is essential too. The Telesurgery collaborative community is working together to address and establish the best practices in the field.

## Introduction

Telesurgery, where surgeons operate remotely on patients located elsewhere, opens doors to enhanced surgical capabilities, wider healthcare reach, and potentially improved patient outcomes. Robotic surgery is the ideal medium through which to perform telesurgery, being initially conceptualized as a tool for providing surgical care in the battlefield at a remote distance. Using robotic surgery as a telesurgical platform was further cemented by the first telerobotic endoscopic surgical procedure, which was almost concomitant to the introduction of commercial robotic surgery dating back to 2001 [1]. After multiple trials with telerobotics in that era, by different institutions (Anvar [2], Nguan [3]), the enthusiasm waded due to increased cost and technology not meeting the requirement for such high equity procedures. Two decades of technological evolution that followed, has allowed the renaissance of of telerobotics. Currently, some new robotic systems entered the market, promising to lower the cost of surgery and enhance the distribution of robotic surgery [4]. Some of these platforms already have intrinsic capability of remote connections. Concomitantly, the last two decades witnessed the evolution of telecommunication, and advances in cellular networks – from 1 to 6G – contributing forward to a fully digital and connected system.

The network reliability is the foundation of successful implementation of telesurgery. It relies on a robust, high-speed communication network, with ultra-low latency. This ensures seamless transmission of real-time video and haptics data between the surgeon’s console and the robot in the operating room. Roundtrip latency, defined as the time delay between the surgeon’s action and his perception of the robot response on his console, is critical. Significant lag has been shown to jeopardize precision and safety during surgery [5, 6]. The effect of different latency has been addressed in an experimental study on the dV-Trainer simulator and evaluated on different tasks: it is recognized that a latency lower than 200 ms is ideal for telesurgery and still acceptable up to 300 ms; at a higher latency, telesurgery becomes tiring and conscious mechanisms of compensation should be adopted to balance the delay [6].

Beyond the latency issue, the full-fledged adoption of telerobotics demands careful consideration of both technical and ethical challenges [7]. A deep insight into these issues has been explored during the first Telesurgery Consensus Conference that took place in Orlando, Florida, USA, on the 3rd and 4th of February, 2024. The event involved all surgical specialties and merged members from scientific societies of robotic surgery, together with international exponents and stakeholders [1].

Data presented during the conference are fully available online and provide an overview of the current status of telerobotic surgery. Herein, a comprehensive summary is delivered to state how telesurgery has been applied so far in human clinical practice.

## Current robotic systems with telesurgery potential

### Hinotori

The Hinotori is a robotic-assisted surgery system developed by Medicaroid, a joint venture between Kawasaki Heavy Industries and Sysmex Corporation, Japan. It is designed as a multiport, single boom system with an immersive console and easily maneuverable surgeon cockpit. Hinotori received its first regulatory approval for urology in Japan in August 2020 from the Japan’s Pharmaceuticals and Medical Devices Agency (PMDA). In October 2022, its use expanded to gynecology and general surgery. Currently, Medicaroid Europe is actively pursuing CE marking compliance under MDR (2017/745) regulations, paving the way for its introduction in the European market. Hinotori was specifically intended to address some limitations of existing surgical robots, such as large size and high cost. Hinotori is smaller in terms of overall footprint and has a wide range of motion, allowing complex procedures. In addition, Hinotori provides an intuitive interface and haptic feedback. It has been used in a variety of surgical procedures, including general surgery, urology, gynecology, thoracic surgery and orthopedic surgery. Two studies comparing Hinotori with the Da Vinci are already available for partial nephrectomy and radical prostatectomy and found no differences in terms of peri-operative outcomes (including positive margin rate, ischemia time for renal surgery) [8, 9].

To date, *Hinotori’s* telesurgical experience has been in preclinical testing only [10, 11]. It operated on animal models, a dry model and cadavers. The distances between the surgeon console and patient/subject while using the Hinotori system ranged between 150 and 2000 km. The connections used were either a dedicated network, guaranteed-type lines (fiber optics) or a 5G wireless network. The distance considered to be the farthest in Japan is 2000 km, from Hokkaido University to Kyushu University. The bandwidth required for telesurgery as well as communication delay and packet loss issues were addressed. A full telesurgical distal gastrectomy on a cadaver was performed over a distance of 250 km. The mean round-trip latency was 40 ms, which is considered safe for telesurgery [11]. To note, Japan has already released Telesurgery Guidelines by the Japanese Surgical Society.

### Edge medical telesurgery system

The core soft tissue robotic platforms from Shenzen Edge Medical Company, Shenzen, China, are the Multiport 1000, which gained the NMPA approval for urology, gynecology, thoracic and general surgery in August 2023, and the Single Port SP1000 that has been NMPA approved for gynecology in November 2023. Both systems are designed with an immersive console and the multiport has a single boom conformation.

The system is designed with telesurgical capabilities and contains built-in high-performance communication modules and low-latency control systems to enable remote surgical procedures [12]. In collaboration with PLA General Hospital, more than one hundred animal and 30 live human surgeries have taken place. Human cases involved urological, gynecological and general surgery procedures. The overall round-trip latency was less than 200 ms in all cases with an almost absent frame loss. A portion of the human experience has been published in European Urology by Wang et al. [12]. Six patients diagnosed with a retrocaval ureter, renal cancer, prostate cancer and an adrenal tumor underwent telesurgery between Beijing and Sanya, with a round-trip communication distance of more than 6000 km. For all procedures, the real-time network latency ranged from 48.37 to 52.20 ms. Total latency including video encoding/decoding latency and robot master–slave latency was between 168.37 and 172.20 ms, with the surgeons reporting no perceivable delay. The case series confirmed the feasibility of performing telesurgery with the Edge MP 1000, at least in the urological setting. To note, the EDGE MP1000 system has been showcased in several live telesurgery events.

### KangDuo

The KangDuo Surgical Robot System (KD-SR-01) is a robotic surgery system developed in China as well, specifically designed for urological procedures. It has an open surgeon console with two HD monitors and the patient cart is designed as a “single boom” with either a 3-arm model (SR 1000, SR. 1500) or a 4-arm one (SR2000). A single arm located on an independent cart is available as well if needed (SR 23110 and SR 3000), allowing for a 5-arm surgery. The laparoscopic arms of the system are compatible with several kinds of endoscopes from different manufacturers (ie Storz, Olympus) and allow the utilization of various accessory equipment. The system has already integrated opportunities for firefly fluorescence imaging, with a 4K HD fluorescent navigation endoscope as well as integrated systems for augmented reality surgical navigation and for the use of laparoscopic and robotic ultrasound probes. Its use has been already reported for robotic-assisted partial nephrectomies and radical prostatectomies. A prospective randomized controlled trial on 100 patients showed non-inferiority of the KangDuo compared to the Da Vinci for partial nephrectomy, regarding safety and efficacy for T1a lesions [13]. A comparison between Da Vinci and KangDuo robot-assisted radical prostatectomy has been recently published and found similar short-term oncologic and functional outcomes with longer operation time using the KangDuo [14]. The system has been used also for colo-rectal surgery [15, 16].

The *KangDuo* robotic system has been used for some human telesurgical operations. The company pursued the concept of multiple consoles for telesurgery, given the risk associated with the use of a sole remote console. In the single console setting, if unexpected technical difficulties occur, the local surgeon is unable to manage the case robotically; thus, the whole procedure is intimately dependent on network conditions. The presence of multiple consoles mitigates the risk of loss of connection between the patient and main remote surgeon console, as well as gives opportunities to perform teaching operations and telementoring. By the end of 2023, some telesurgical cases have been performed either on animal or human subjects. Animal experiments included gastrointestinal and liver resection surgeries with surgeons located in Suzhou and Beijing, with a distance of more than 1000 km. The system enables also the connection of three consoles to achieve a Triple-Console Telesurgery, ideal for training purposes. Human procedures have been already performed by Prof. Patel, with one console located in Beijing, another in Hainan (3000 km) and the third located in Changsha (Hunan) where the patient is located. The open console of the KangDuo with three screens enables the remote surgeon to visualize the operating room in real-time. The connections that were tested in different surgeries included 5G wireless networks as well as wired fiber optic ones.

### Microport Medbot

The MicroPort MedBot Robotic Systems is a collection of several robotic-assisted surgery systems developed by the Chinese company MicroPort Robot (Zhuhai) Co., Ltd. The company provides several key systems for orthopedic, pulmonary and prostate biopsy indications as well as soft tissue surgery. The Toumai laparoscopic surgical system is the one NMPA approved for abdominal surgery with the CE approval already in progress. It is designed with an immersive console and a single boom, multiport patient cart. It allows urological, gynecological, thoracic and general surgery procedures.

The *Toumai from Microport* is the system with the largest series on telesurgery in human procedures. The Toumai is designed to be compatible with multiple networks including 5G wireless networks. Moreover, it is capable of dual console operation, with the consoles being located both at the patient’s site and remotely, or entirely remote. The first telesurgical case was performed on an ultra-long distance of almost 5000 km (Shanghai-Xinjiang) by using a 5G network, and deroofing of a renal cyst was performed with a latency > 310 ms. Thereafter, multiple procedures have been performed exceeding 100 + human cases. General surgery accounted for 61%, urology for 32% and gynecology for 7% of the cases. Complex indications included radical gastrectomy, radical prostatectomy, liver resection, radical nephrectomy, pancreatectomy, bariatric surgery and hysterectomy. Networks that were used were either 5G or dedicated ground lines. Within a range of 200 km between surgeon and patient, the average delay is 24 and 41 ms with a dedicated line and 5G/LAN, respectively. As the range increases to between 1000 and 2000 km, the integration of 5G and LAN enabled an average delay of 52 ms, whereas at a distance of 5000 km the dedicated line and the 5G/LAN connections allowed an average delay of 73 and 159 ms, respectively. Among the Toumai telesurgery procedures, two cases of robot-assisted laparoscopic spermatic vein ligation have been published [17]. The surgeries were performed at Xinjiang Kezhou People’s Hospital, with the surgeon’s console in the Telemedical Center of the First Affiliated Hospital of Nanjing Medical University at a linear distance of about 3 800 km. The interventions were performed using the public 5G network. The intraoperative average round-trip network delay was 130 ms, and the average continuous data packet loss rate was 1.4%. No adverse network events, such as network interruption occurred, confirming the safety of telesurgery with the system. The Toumai has also been used in surgical simulation at a distance that has reached over 12,500 km (Orlando – Dubai).

## Conclusion

During the Telesurgery meeting in Orlando in February 2024, representatives and experts from leading robotic surgical device developers delivered insightful presentations, describing collaborative ventures with hospitals, transitioning from pre-clinical investigations to telesurgery procedures using advanced 5G and fiber optic communication technologies, thus advancing routine clinical practice. A unanimous consensus emerged among all participants, affirming the growing significance of telemonitoring and telepresence in robotic surgery, signaling a crucial evolutionary step toward broader global adoption of telesurgery, minimizing costs, and increasing potential.

Beyond technical network specifications, addressing cybersecurity, data privacy, and redundancy in technical malfunctions is imperative for successful telesurgery implementation. Expanding the discussion to encompass ethical, financial, regulatory, and legal considerations is also essential before telesurgery can be adopted within the robotic surgical community. In this scenario, our Telesurgery collaborative community is working together to address and establish the best practices in the field (Table 1).

**Table 1:** A summary of clinical telerobotic applications performed so far

Robot	System	Characteristics	Application	N	Connection	Average delay	Max distance
Hinotori	Medicaroid, Japan	Single boom, multiport	Animal,lab cadaver		Dedicated network, 5G, guaranteed-type line	–	–
MPlOOO	Edge Medical, System,China	Single boom, multiport	Human	30	Dedicated line, China Telecom	< 200 ms	3000 km
SP 1000		Single boom, single port					
Kangduo	Kangduo Robotics, China	Single boom (+ 1additional arm), open console, dual/triple console	Human	Live human	5G, wired networks	–	3000 km
Toumai	Microport, Medbot, China	lmmersive console, multiport	Human	100 + . *	5G, dedicated network, internet	24–41 ms at 200 km; 52 ms at 1000–2000 km; up to 159 ms at 5000 km	5000 km

*Toumai: General Surgery cases 61%,Urology 32%,Gynecology 7%

## Data Availability

No datasets were generated or analysed during the current study.
